# Effectiveness of XP-Endo Finisher, Endoactivator, and PUI agitation in the penetration of intracanal medicaments into dentinal tubules: A confocal laser scanning microscope analysis

**DOI:** 10.34172/joddd.41123

**Published:** 2024-09-07

**Authors:** Elif Akkol, Esin Özlek

**Affiliations:** ^1^Sancaktepe Oral and Dental Health Center, Istanbul, Turkey; ^2^Department of Endodontics, Faculty of Dentistry, University of Van Yuzuncu Yıl, Van, Turkey

**Keywords:** Confocal laser scanning microscopy, Dentinal tubules, Irrigation activation systems, Root canal medicaments, Root canal preparation

## Abstract

**Background.:**

This investigation assessed the impact of irrigation activation systems on the depth of penetration of intracanal medicaments into dentinal tubules.

**Methods.:**

Ninety-six mandibular premolar teeth were prepared using ProTaper Next up to size X3. The teeth were randomly divided into four groups based on the final irrigation activation systems (n=24): group 1: EndoActivator, group 2: XP-Endo Finisher, group 3: Passive ultrasonic irrigation (PUI), and group 4: control. After the final irrigation, all the samples were divided into two subgroups (n=12): subgroup A: calcium hydroxide (Ca(OH)_2_ ) and subgroup B: modified triple antibiotic paste (mTAP). Statistical analysis involved three-way analysis of variance (ANOVA).

**Results.:**

EndoActivator, XP-Endo Finisher, and PUI activation methods significantly increased the penetration of intracanal medicaments compared to conventional needle irrigation (*P*<0.05). The XP-Endo Finisher group exhibited the highest penetration percentage and maximum penetration depth, showing a statistically significant difference from the EndoActivator group (*P*<0.001). No significant difference was observed between XP-Endo Finisher and PUI, nor between PUI and EndoActivator (*P*>0.05). mTAP showed a higher percentage of dentinal tubule penetration than Ca(OH)_2_, although no significant difference was found in maximum penetration depth. The coronal region demonstrated the highest penetration percentage and depth, while the apical region showed the lowest.

**Conclusion.:**

This study showed the effectiveness of XP-Endo Finisher in improving the penetration of intracanal medicaments into dentinal tubules. The findings emphasize the importance of selecting appropriate irrigation activation systems to improve treatment outcomes in endodontics. By demonstrating the effectiveness of advanced systems like XP-Endo Finisher, this research supports their integration into routine clinical practice for better endodontic success.

## Introduction

 Intracanal medicaments in endodontic treatments are crucial in eliminating microorganisms from the root canals and controlling infections. The deep penetration of these medicaments into dentinal tubules is essential for enhancing treatment success. Insufficient penetration can lead to residual microorganisms, resulting in recurrent infections.^1^

 However, the complex morphological structure of root canals and the presence of a smear layer can hinder the effective penetration of medicaments into dentinal tubules. The smear layer acts as a barrier on the root canal surface, making it difficult for medicaments to penetrate deeply. Traditional irrigation methods may be inadequate in thoroughly cleaning the root canal and removing the smear layer completely. Various irrigation activation systems have been developed in recent years to overcome these limitations. These systems enhance the efficacy of irrigation solutions, allowing for more thorough cleaning of the root canals and improved penetration of medicaments into dentinal tubules.^2,3^

 The EndoActivator (Dentsply, Tulsa, OK, USA) is a sonic irrigation system employing a portable micromotor and durable polymer tips of various sizes. These flexible tips, resistant to breakage, move in an apicocoronal direction, creating a robust hydrodynamic effect for enhanced solution penetration in challenging root canal areas.^4,5^ Passive ultrasonic irrigation (PUI) utilizes ultrasonically activated stainless steel files, generating energy through horizontal vibrations. This method induces multiple acoustic stressors, improving solution movement within the root canal and proving more effective in debris removal than the conventional syringe method.^6^ The XP-Endo Finisher (FKG Dentaire SA, Switzerland) is a MaxWire alloy file system designed for irrigation activation. According to the manufacturer, its unique property involves transitioning from the martensitic phase (M) to the austenite (A) phase within the root canal, ensuring irrigation without dentin damage in rotation mode.^7^

 While a limited number of studies have evaluated the dentinal tubule penetration of intracanal medicaments,^8,9^ this study assessed the effects of intracanal medicaments (Ca(OH)_2_ and mTAP) using different final irrigation activation systems (XP-Endo Finisher, EndoActivator, and PUI) on dentinal tubule penetration using confocal laser scanning microscopy (CLSM). The null hypothesis of this study is that different irrigation activation systems (XP-Endo Finisher, EndoActivator, and PUI) have no significant effect on the penetration of intracanal medicaments into dentinal tubules.

## Methods

 Single-rooted mandibular premolars (n = 96) with completely formed roots and closed apices were collected based on a protocol approved (2019/03-05) by the Research Ethics Board of the University. The sample size was calculated using the G*Power program (3.1.9.2; Heinrich Heine University, Düsseldorf, Germany) based on Cohen’s criteria for effect size. With a 5% type 1 alpha error (α = 0.05), 75% power (1-β = 0.75), and a large effect size (f = 0.40), the required minimum sample size was 96. Each tooth was meticulously examined under a stereomicroscope (Nikon SMZ25; Nikon Tokyo, Japan), with exclusion criteria for exhibiting cracks or fractures. Subsequently, radiographs were taken in the mesiodistal and buccopalatal direction to verify the single-canal nature of the teeth. Inclusion criteria encompassed teeth with no calcification in the root canal, a curvature not exceeding 10⁰, no evidence of root resorption, and a root length of at least 15 mm. The curvature of the root canals was measured using Schneider’s method. This method involves taking a standardized radiograph of the tooth and drawing two lines: one from the orifice to the apex along the long axis of the canal and another from the apex to the point where the canal deviates from the original line. The angle formed between these two lines represents the curvature of the canal. All the measurements were made using the ImageJ program (ImageJ, 1.51s, National Institutes of Health, Bethesda, MD, USA) to ensure accuracy. After removing soft and hard tissue deposits using a curette, the teeth were stored in distilled water at room temperature until use. The root lengths were standardized to 12 mm by removing the tooth crowns with a diamond separator under water cooling. All the specimens were instrumented up to X3 by a single operator with the ProTaper Next (Dentsply, Maillefer, Ballaigues, Switzerland) rotary file system. During instrumentation, 2 mL of 5.25% sodium hypochlorite (NaOCl) solution (Microvem AF, Istanbul, Turkey) was used as the irrigation solution at each file change. For the final irrigation, 5 mL of 17% ethylenediaminetetraacetic acid (EDTA) (Imicrly, Konya, Turkey) and 5 mL of 5.25% NaOCl solution were used. All irrigation procedures were performed using a 31-gauge closed-end needle (Ayset, Adana, Turkey). Then, all samples were randomly divided into four groups (n = 24) according to the final irrigation activation system using a computer program (https://www.random.org/):

- Group 1 (EndoActivator):Following the introduction of the final irrigation solution into the root canal, the red tip (25/04) of the EndoActivator (Dentsply, Tulsa, OK) was positioned 1 mm shorter than the working length. Activation was performed for three periods of 20 seconds each, totaling 1 minute, at a frequency of (10 000 cpm).
- Group 2 (XP-Endo Finisher):After introducing the final irrigation solution into the root canal, the XP-Endo Finisher (FGK Dentaire, La Chaux-de-Fonds, Switzerland) file was employed for activation for three periods of 20 seconds, totaling 1 minute. The XP-Endo Finisher file was positioned 1 mm shorter than the working length and operated with an X-Smart Plus (Dentsply, Maillefer, Ballaigues, Switzerland) endodontic motor at 800 rpm with a torque of 1 Ncm following the manufacturer’s instructions.
- Group 3 (PUI):After placing the final irrigation solution in the root canal, an ultrasonic device was used for activation for three periods of 20 seconds each, totaling 1 minute. The ultrasonic device (VDW, Munich, Germany) was activated by placing the 21-mm IRR20 irrigation tip 1 mm shorter than the working length and setting the power to 30.
- Group 4 (control group): After placing the final irrigation solution in the root canal, activation was achieved by moving the 0.4 mm diameter 30-gauge dental needle tip in the apicocoronal direction for 1 minute.

All the root canals were irrigated with 2 mL of distilled water to eliminate the long-term effects of EDTA and NaOCl solutions, followed by drying with absorbent paper points. Subsequently, all the samples were randomly divided into two subgroups according to the intracanal medicament to be used: *subgroup A:* calcium hydroxide (Kalsin, Aktu Tic, İzmir, Turkey); *subgroup B:* mTAP (modified triple antibiotic paste) – composed of ciprofloxacin (Cipro 500 mg; Biofarma Pharm Ind Ltd, Istanbul, Turkey), metronidazole (Flagyl 500 mg; Sanofi Aventis Pharm Inc Co, Istanbul, Turkey), and cefaclor (Sanocef 750 mg film tablet, Actavis, Istanbul, Turkey), prepared by mixing with distilled water at a 1:1:1 ratio.^8^

 Both medicaments (Ca(OH)_2_ and mTAP) in both subgroups were blended with 0.1% Rhodamine B fluorescent substance (Merck Chemistry).^10^ The stability of the dye mixed in the sealer was verified and confirmed in pilot studies. Root canal medicaments were applied with a #30 lentulo (Dentsply, Maillefer, Switzerland) attached to an endodontic motor, operated at 800 rpm until visible from the apical root canal. The access cavities of the teeth were sealed with cotton pellets and temporary filling material Cavit-G (3M-ESPE, Minnesota, USA), and the samples were incubated at 37 °C and 100% humidity for 24 hours.

###  Confocal laser scanning microscope analysis

**Figure 1:**
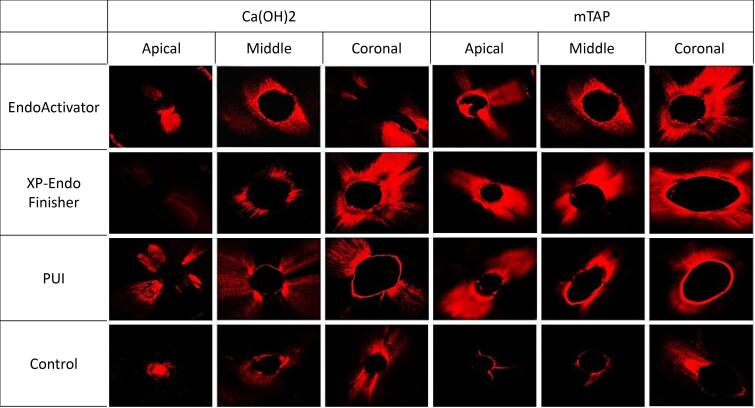
Representative confocal laser scanning microscope images from each group

**Figure 2:**
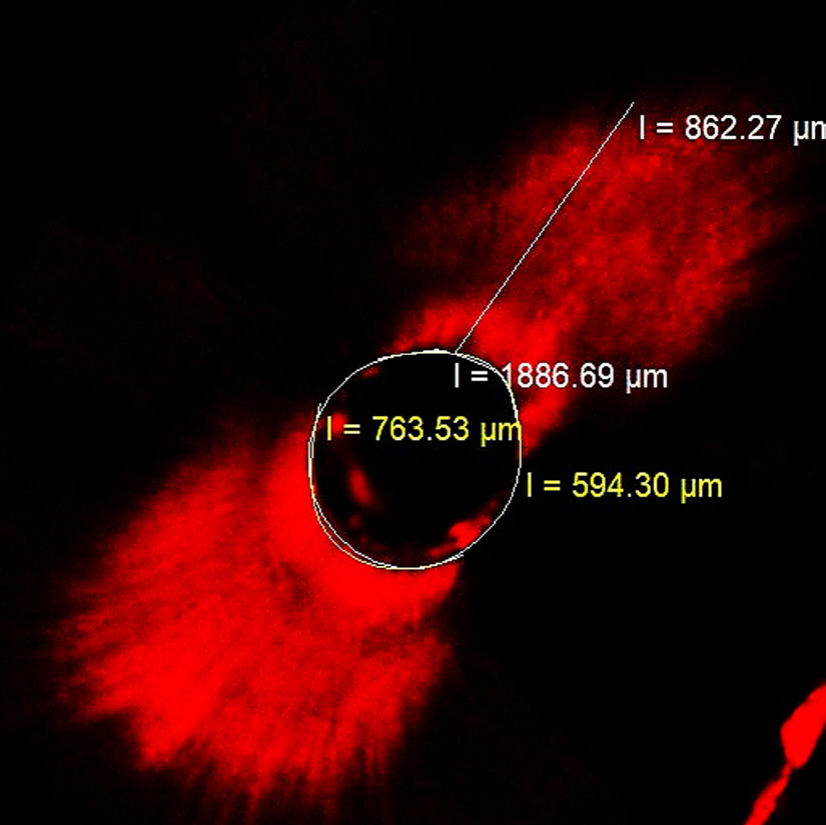
A confocal laser scanning microscopic image showing the percentage and maximum penetration depth measurement using the NIS-Elements Microscope Imaging software

 All the samples were embedded into acrylic blocks and horizontally sectioned using an IsoMet saw (IsoMet, Buehler, Lake Bluff, IL, USA) to obtain 1-mm-thick sections from 2-, 5-, and 8-mm levels from the apex. A total of 288 sections were obtained and imaged at × 4 magnification using a confocal laser microscope. These images were then transferred to the Zeiss LSM Image Browser v.4.2.0 program. Representative images from each group are shown in Figure 1. Dentinal tubule penetration percentages and maximum penetration depths were quantified using the program’s image tools. The percentage of dentinal tubule penetration was calculated by dividing the sum of the length of the regions where intracanal medicaments penetrate the root canal wall by the root canal wall perimeter.^11^ The maximum penetration depth was determined by measuring the distance between the root canal wall and the furthest penetration point (Figure 2).^12^ The investigator who evaluated the images was blinded to the group assignments to prevent bias in assessing the results.

###  Statistical analysis

 The data were analyzed with IBM SPSS 23. Normal distribution was assessed through the Shapiro-Wilk test. A three-way analysis of variance (ANOVA) was employed to compare mean values of dentinal tubule penetration percentages and maximum penetration depths based on final irrigation activation methods, intracanal medicaments, and root canal regions. A significance level of *P* < 0.05 was considered statistically significant.

## Results

 Three-way ANOVA revealed that the final irrigation activation systems significantly influenced the percentage of dentinal tubule penetration of intracanal medicaments and root canal regions (*P* < 0.001) (Table 1). Additionally, both the final irrigation activation systems and root canal regions significantly affected the maximum penetration depth (*P* < 0.001) (Table 2).

**Table 1:** Three-way ANOVA for the final irrigation activation system, intracanal medicament, root canal region, and the interaction terms according to the dentinal tubule penetration percentage

**Source of variation**	**Type III sum of** **squares**	**SD**	**Mean square**	***F***	**Sig.**
Final irrigation activation system	16511	3	5503.7	21.2	< 0.001
Intracanal medicament	8103	1	8103	31212	< 0.001
Root canal region	19410.2	2	9705.1	37.383	< 0.001
Final irrigation activation system * Intracanal medicament	260.1	3	86.7	0.334	0.801
Final irrigation activation system * Root canal third	1135.9	6	189.3	0.729	0.626
Intracanal medicament * Root canal region	1276.2	2	638.1	2.458	0.088
Final irrigation activation system * Intracanal medicament* Root canal region	829.2	6	138.2	0.532	0.783

Statistically significant difference at *P* < 0.05.

**Table 2:** Three-way ANOVA for the final irrigation activation system, intracanal medicament, root canal region, and the interaction terms according to the maximum penetration depth

**Source of variation**	**Type III sum of** **squares**	**SD**	**Mean square**	***F***	**Sig.**
Final irrigation activation system	11849601.9	3	3949867.3	30.326	< 0.001
Intracanal medicament	544.7	1	544.7	0.004	0.948
Root canal region	8757363	2	4378681.5	33.619	< 0.001
Final irrigation activation system * Intracanal medicament	433693.4	3	144564.5	1.11	0.346
Final irrigation activation system * Root canal third	247795.7	6	41299.3	0.317	0.928
Intracanal medicament * Root canal region	1365641.1	2	682820.5	5.243	0.006
Final irrigation activation system * Intracanal medicament* Root canal region	608365.4	6	101394.2	0.778	0.587

Statistically significant difference at *P* < 0.05.

 Irrespective of root canal regions and the type of intracanal medicament, different final irrigation activation systems demonstrated a statistically significant impact on the dentinal tubule penetration percentage and maximum penetration depth of the medicaments (*P* < 0.001). The XP-Endo Finisher group exhibited the highest penetration percentage and maximum penetration depth. While no statistically significant difference was observed between the XP-Endo Finisher and PUI activation groups for both parameters (*P* > 0.05), the XP-Endo Finisher group was significantly more effective than the EndoActivator group (*P* < 0.001). No statistically significant difference was noted between PUI and EndoActivator for both parameters (*P* > 0.05). Three-way ANOVA also indicated a statistically significant effect of the final irrigation activation systems on the percentage of dentinal tubule penetration of intracanal medicaments and root canal regions (*P* < 0.001) (Table 1). Both the final irrigation activation systems and root canal regions significantly influenced the maximum penetration depth (*P* < 0.001) (Table 2). Regardless of the final irrigation systems and root canal regions, the type of intracanal medicament significantly affected the percentage of dentinal tubule penetration (*P* < 0.001) (Table 3). mTAP exhibited a higher percentage of dentinal tubule penetration than Ca(OH)_2_. However, there was no statistically significant difference between intracanal medicaments regarding maximum penetration depth *(P* = 0.948).

**Table 3:** Multiple comparisons of dentinal tubule penetration percentages

**Final irrigation activation system**	**Intracanal medicament**	Coronal*	Middle*	Apical*	Total*
EndoActivator	Ca(OH)_2_	69.1 ± 13.7	68.3 ± 10.1	43 ± 18.8	60.1 ± 18.8
	mTAP	76.1 ± 11.5	75.3 ± 10.5	57 ± 18.7	69.5 ± 16.3
	Total	72.6 ± 12.9	71.8 ± 10.7	50 ± 19.7	64.8 ± 18.1^c^
XP-Endo Finisher	Ca(OH)_2_	77.0 ± 14.8	68.3 ± 19.2	51.2 ± 15	65.5 ± 19.3
	mTAP	83.8 ± 10	76.9 ± 13.7	75.9 ± 16.4	78.9 ± 13.7
	Total	80.4 ± 12.9	72.6 ± 16.9	63.6 ± 19.9	72.2 ± 17.9^b^
PUI	Ca(OH)_2_	67.4 ± 13.3	68.2 ± 16.9	49 ± 25.4	61.5 ± 20.7
	mTAP	82.5 ± 15.5	71.8 ± 17.4	64.2 ± 22.7	72.8 ± 19.7
	Total	75.0 ± 16.1	70 ± 16.9	56.6 ± 24.8	67.2 ± 20.9^bc^
Control	Ca(OH)_2_	59.2 ± 12,4	48.3 ± 12.6	34.8 ± 14.5	47.5 ± 16.3
	mTAP	63.0 ± 20,0	57.6 ± 10.3	47.1 ± 20.8	55.9 ± 18.5
	Total	61.1 ± 16,4	53 ± 12.2	41 ± 18.6	51.7 ± 17.8^a^
Total	Ca(OH)_2_	68.2 ± 14.6	63.3 ± 17.0	44.5 ± 19.4	58.7 ± 19.8
	mTAP	76.4 ± 16.6	70.4 ± 15.0	61 ± 21.9	69.3 ± 19.0
	Total	72.3 ± 16.1^a^	66.8 ± 16.3^a^	52.8 ± 22.2^b^	64 ± 20.1

a-c: No difference between times with the same letter within groups. * mean ± standard deviation.

 Significant differences were observed between dentinal tubule penetration percentages and maximum penetration depths of intracanal medicaments in different root canal regions (*P* < 0.001). The apical region exhibited the lowest dentinal tubule penetration percentage and the maximum penetration depth, while no significant difference was found in the percentage of dentinal tubule penetration between the coronal and middle regions (*P* > 0.05). However, the coronal region showed a significantly higher maximum penetration depth (*P* < 0.001) (Table 4).

**Table 4:** Multiple comparisons of maximum penetration depths

**Final irrigation activation system**	**Intracanal medicament**	Coronal*	Middle*	Apical*	Total*
EndoActivator	Ca(OH)_2_	1373.5 ± 311.7	1246.2 ± 268.5	804.1 ± 314.1	1141.1 ± 381.3
	mTAP	1144.4 ± 283.2	1051.9 ± 221	842.8 ± 406.5	1013 ± 330
	Total	1258.7 ± 313.8	1149.1 ± 260.1	823.4 ± 355.8	1077.1 ± 359.8^b^
XP-Endo Finisher	Ca(OH)_2_	1508.5 ± 550.6	1065.8 ± 481.5	778.6 ± 413.3	1117.6 ± 560.8
	mTAP	1224.7 ± 323.7	1213.1 ± 417.6	1138.2 ± 398.9	1192 ± 373.2
	Total	1366.6 ± 464.9	1139.4 ± 447.1	958.4 ± 437.7	1154.8 ± 474.5^b^
PUI	Ca(OH)_2_	1242.6 ± 492.1	1183.8 ± 342.6	761.8 ± 420.4	1062.7 ± 464.4
	mTAP	1209.9 ± 396	1124.5 ± 390.8	842 ± 337.7	1058.8 ± 398.2
	Total	1226.3 ± 437.1	1154.2 ± 360.7	801.9 ± 375.1	1060.8 ± 429.5^b^
Control	Ca(OH)_2_	846.8 ± 370	704.3 ± 302.3	288 ± 198.7	613 ± 376.8
	mTAP	794.3 ± 290.2	648.9 ± 219.4	535.9 ± 244.1	659.7 ± 268
	Total	820.6 ± 326.3	676.6 ± 259.9	411.9 ± 251.8	636.4 ± 325.5^a^
Total	Ca(OH)_2_	1242.7 ± 494,8^C^	1050 ± 406.1^BC^	658.1 ± 400.7^A^	983.6 ± 497
	mTAP	1093.3 ± 362^C^	1009.6 ± 383.2^BC^	839.7 ± 403.6^AB^	980.9 ± 395.1
	Total	1168 ± 437.7^a^	1029.8 ± 393.3^b^	748.9 ± 410.3^c^	982.3 ± 448.2

a-c: No difference between times with the same letter within groups. * mean ± standard deviation.

## Discussion

 Intracanal medicaments are frequently used in multi-session root canal treatments to eliminate microorganisms that persist in the root canal between sessions. These medications must remain in contact with the root canal walls for an extended duration and penetrate deeply into the dentinal tubules to be effective.^8,9^ This study assessed the impact of intracanal medicaments (Ca(OH)_2_/mTAP) used with different final irrigation activation systems (EndoActivator, XP-Endo Finisher, and PUI) on dentinal tubule penetration percentage and maximum penetration depth. The results indicated a statistically significant influence of final irrigation activation systems on the dentinal tubule penetration percentage and maximum penetration depth of intracanal medicaments.

 De Oliveira et al^13^ reported the significant impact of sonic activation (EndoActivator) on the dentinal tubule penetration percentage and maximum penetration depth of root canal sealers (*P* < 0.05). Bolles et al^14^ assessed the effect of final irrigation with EndoActivator, Vibringe, and the conventional syringe method on the dentinal tubule penetration percentage and maximum penetration depth of root canal sealer, indicating no statistically significant difference between the final irrigation activation systems (*P* > 0.05). On the contrary, Uroz-Torres et al^15^ compared the effectiveness of irrigation activation with EndoActivator with that of conventional syringe irrigation in removing the smear layer and reported no statistically significant difference between the two methods (*P* > 0.05). Generali et al^11^ evaluated the effect of different irrigation activation systems (EndoActivator, Irrısafe, Self-Adjusting File (SAF), and EndoVac) on the dentinal tubule penetration percentage and maximum penetration depth of root canal sealer, reporting no statistically significant difference between the groups (*P* > 0.05). Regarding dentinal tubule penetration percentage and maximum penetration depth (*P* < 0.05), the final irrigation activation with EndoActivator was significantly more effective than the conventional syringe method. Bolles et al^14^ used a total irrigation activation time of 1 minute with EndoActivator, while Generali et al^11^ used a total irrigation activation time of 2 minutes in their study. In this study, the longer irrigation activation times compared to the other two studies possibly contributed to the enhanced effectiveness of EndoActivator. Consequently, the EndoActivator irrigation group was significantly more effective than the conventional syringe method (*P* < 0.05).

 Leoni et al^16^ assessed the effectiveness of different final irrigation activation systems (PUI, XP-Endo Finisher, and SAF) in debris removal using micro-CT. The results indicated that irrigation activation with PUI and XP-Endo Finisher files removed debris more effectively than SAF, with no significant difference between PUI and XP-Endo Finisher. Elnaghy et al^17^ evaluated the effectiveness of final irrigation activation with the EndoActivator, XP-Endo Finisher, and BT-Race rotary file on debris and smear layer removal from curved root canals. The study reported that irrigation activation with the XP-Endo Finisher file system and the Endo Activator with BT-Race was significantly more effective than the rotary file system. Pacheco-Yanes et al^18^ compared the effectiveness of the NaOCl solution of the PUI and XP-Endo Finisher file system on dentinal tubule penetration, reporting that the XP-Endo Finisher file system was significantly more effective. Turkaydın et al^19^ studied the effectiveness of PUI and the XP-Endo Finisher file system in removing triple antibiotic paste from the root canal and reported that the XP-Endo Finisher file system was significantly more effective. According to recent studies, researchers have reached a consensus that activation with the XP-Endo Finisher file system is more effective than other methods. The enhanced effectiveness of the XP-Endo Finisher file is attributed to its ability to enter the A phase when placed in the root canal, and its shape allows it to touch the dentin surface more effectively in this phase, affecting areas that cannot be reached with standard files.^17^ The results of this study can be compared to those of existing studies in the literature. Intracanal medicaments and dentinal tubules, from which debris and smear layer have been removed, are expected to allow for better penetration. According to the findings of this study, intracanal medicaments demonstrated deeper penetration with final irrigation using PUI, EndoActivator, and XP-Endo Finisher file compared to the conventional syringe method (*P* < 0.05). The dentinal tubule penetration percentage and maximum penetration depth values of the medicaments were higher when the XP-Endo Finisher file system was used. These results align with the available literature.

 Faria et al^20^ compared the effect of PUI and conventional syringe irrigation on the penetration of NaOCl solution into the dentinal tubules; activation with PUI was significantly more effective than with conventional syringe irrigation. De-Deus et al^21^ compared the debris removal efficiency of irrigation activation with PUI and XP-Endo Finisher, reporting both activation methods as equally effective. Wiseman et al^22^ compared the efficacy of irrigation activation with PUI and EndoActivator in removing calcium hydroxide, revealing that the ultrasonic system was more effective than the sonic system (*P* < 0.001). According to Sáinz-Pardo et al,^23^ activation with EndoActivator and PUI allowed irrigation solutions to penetrate deeper into the dentinal tubules than the conventional syringe irrigation method. However, no statistically significant difference was observed between the two groups. The XP-Endo Finisher group exhibited the highest penetration percentage and maximum penetration depth in this study. While no statistically significant difference was observed between XP-Endo Finisher and PUI, as well as PUI and EndoActivator for both parameters (*P* > 0.05), the XP-Endo Finisher group was significantly more effective than the EndoActivator group (*P* < 0.001). These findings are consistent with numerous studies in the literature.

 Since bacteria in the root canal system can settle to a depth of 1000 µm in the dentinal tubules, intracanal medicaments must be able to penetrate deeply into the dentinal tubules to affect these bacteria.^24^ Only one study in the literature has evaluated the dentinal tubule penetration of TAP and Ca(OH)_2_ paste. Deniz Sungur et al^8^ investigated the effect of placing TAP and Ca(OH)_2_ in the root canal with different carriers on dentinal tubule penetration. TAP was found to penetrate a wider area than Ca(OH)_2_, regardless of the carrier, but they reported no significant difference in dentinal tubule penetration percentages and maximum penetration depths. Thanks to this high penetration area, they concluded it is more effective on bacteria than Ca(OH)_2_. In our study, mTAP showed a higher penetration percentage than Ca(OH)_2_, regardless of the final irrigation activation system and cross-sectional level. At the same time, no statistically significant difference was found between the two medicaments in maximum penetration depth values. The results of our study are consistent with the only study in the literature.

 According to the findings of this study, regardless of the type of intracanal medicament and the final irrigation activation system, medicaments showed the lowest penetration percentage and maximum penetration depth values in the apical section. In contrast, they showed the highest values in the coronal section, which were significantly different (*P* < 0.05). These findings align with the results of other studies evaluating dentin tubule penetration.^10,14^ The endodontic materials exhibit less penetration in the apical region because of the low number of dentinal tubules in the apical region, the irregular structure of the secondary dentin, and the presence of cement-like tissue in the root canal wall.^25^ Additionally, it is thought that materials can penetrate the dentinal tubules in the apical region less due to the better removal of the smear layer in the coronal region and the irrigation solutions being sent to the coronal region more effectively than the apical region.^16^

 This study has several limitations. First, it was conducted under in vitro conditions, which may not fully simulate the complexities of the clinical environment. Second, the sample size was limited, which might affect the statistical power and the generalizability of the findings. Third, the study used only straight root canals, and the results may differ in curved root canals. Future research should address these limitations by including a larger sample size, evaluating teeth with different curvatures, and conducting clinical studies to validate the findings.

## Conclusion

 The XP-Endo Finisher, EndoActivator, and PUI irrigation activation systems significantly enhanced the penetration of intracanal medicaments into dentinal tubules compared to conventional needle irrigation. Among these, XP-Endo Finisher showed the highest penetration percentage and depth for mTAP and Ca(OH)_2_. Notably, the coronal region exhibited higher penetration than the apical region. Further research is needed to explore the long-term implications and clinical applications of these findings in endodontics.

## Acknowledgments

 This research received no specific grant from funding agencies in the public, commercial, or not-for-profit sectors.

## Competing Interests

 All authors declare that they have no conflicts of interest.

## Ethical Approval

 All procedures performed in studies involving human participants were in accordance with the ethical standards of the institutional and/or national research committee and with the 1964 Helsinki Declaration and its later amendments or comparable ethical standards. The study was approved by the Institutional Review Board and the Ethics Committee of the University (2019/03-05).

## Informed Consent

 All the patients signed an informed consent form after being informed about the objectives, procedures, benefits, and potential risks of the study.
